# Sputum induction: review of literature and proposal for a protocol

**DOI:** 10.1590/S1516-31802003000500008

**Published:** 2003-09-01

**Authors:** Marcos Eduardo Scheicher, João Terra, Elcio Oliveira Vianna

**Keywords:** Sputum, Asthma, Cytological techniques, Tuberculosis, Cystic fibrosis, Escarro, Asma, Técnicas citológicas, Tuberculose, Fibrose cística

## Abstract

Since the 1980s, sputum induction by inhalation of hypertonic saline has been successfully used for diagnosing Pneumocystis carinii pneumonia in patients infected with HIV. In recent years, sputum induction and its subsequent processing has been refined as a noninvasive research tool providing important information about inflammatory events in the lower airways, and it has been used for studying various illnesses. In asthma, one application is to use sputum inflammatory indices to increase our understanding of complex relationships between inflammatory cells, mediators, and cytokine mechanisms. In chronic obstructive pulmonary disease, sputum assessment could be used as a screening test before deciding on long-term corticosteroid treatment. In tuberculosis, sputum induction is a valuable diagnostic tool for HIV-seropositive patients who do not produce sputum. Sputum induction appears to be a relatively safe, noninvasive means of obtaining airway secretions from subjects with cystic fibrosis, especially from those who do not normally produce sputum. Moreover, sputum induction can also be used in chronic cough and lung cancer. Generally, induction is performed through ultrasonic nebulizers, using hypertonic saline. It is recommended that sputum be processed as soon as possible, with complete homogenization by the use of dithiothreitol. We have also shown in this article an example of a protocol for inducing and processing sputum employing a nebulizer produced in Brazil.

## RESUMO

Desde a década de 80, a indução de escarro pela inalação de solução salina hipertônica tem sido usada com sucesso para diagnosticar pneumonia por *Pneumocystis carinii* em pacientes infectados pelo HIV. Em anos recentes, a indução de escarro e seu subseqüente processamento foram refinados como uma ferramenta de pesquisa não invasiva que fornece informações importantes sobre eventos inflamatórios nas pequenas vias aéreas, sendo usada para estudar várias doenças. Na asma, uma aplicação é para o uso de índices inflamatórios do escarro para aumentar nossa compreensão de complexos relacionamentos entre célula inflamatória, mediador e mecanismos das citocinas. Na doença pulmonar obstrutiva crônica, a avaliação do escarro pode ser usada como um teste de seleção antes de se decidir pelo tratamento a longo prazo com corticosteróide. Na tuberculose, a indução do escarro é uma ferramenta valiosa para o diagnóstico nos pacientes HIVsoropositivos que não produzem escarro. A indução de escarro parece ser um meio relativamente seguro, não-invasivo de se obter secreções das vias aéreas de sujeitos com fibrose cística, especialmente aqueles que não produzem normalmente o escarro. Além disso, a indução do escarro também pode ser usada na tosse crônica e no câncer de pulmão. Geralmente, a indução é executada através dos nebulizadores ultra-sônicos, usando solução salina hipertônica. Recomenda-se que o escarro seja processado o mais cedo possível, com homogeneização completa pelo uso de ditiotreitol. Mostramos também neste artigo um exemplo de protocolo que emprega um nebulizador de fabricação nacional para induzir o escarro.

## INTRODUCTION

Since the 1980s, sputum induction by inhalation of hypertonic saline has been successfully used for diagnosing *Pneumocystis carinii* pneumonia in patients infected with HIV. Pitchenik et al. (1986) showed that, with 5% hypertonic saline administered via an ultrasonic nebulizer for 10 or 20 minutes, sputum could be induced in the majority of patients with aids and in patients with *Pneumocystis carinii* pneumonia.^1^ Pin et al. (1992) adapted the method for use in asthmatic subjects, and this was the first study to attempt to use induced sputum for examining the inflammatory response in asthma.^2^ In recent years, sputum induction by hypertonic saline and its subsequent processing has been refined as a noninvasive research tool providing important information about inflammatory events in the lower airways. In conclusion, the ability to study inflammation has changed considerably with the development of this technique as a research tool and increasingly as a clinical tool.^3^ Induced sputum has been used for studying various illnesses: asthma, chronic obstructive pulmonary disease, tuberculosis, *Pneumocystis carinii* pneumonia, cystic fibrosis, lung cancer and chronic cough.

Induced sputum has several advantages over other techniques. Bronchoscopy is an invasive procedure and is not easily applicable on a large scale in follow-up studies. Sputum analysis might be an alternative to this, for obtaining airway secretions that may potentially be used for monitoring airway inflammation.^4^ Although fiberoptic bronchoscopy with trans-bronchial biopsy, bronchial brushing and bronchoalveolar lavage are relatively safe procedures, they still entail some morbidity and are relatively unpleasant and expensive procedures compared with sputum induction.^1^ With sputum induction, samples can be obtained from the lower airways with minimal discomfort to the patient. Bronchoscopy allows sampling of the cells and mediators in the airway lumens by means of bronchoalveolar lavage and enables biopsy of the mucosal tissue. The combined information thus obtained is therefore superior to that of sputum alone. However, bronchoalveolar lavage samples only distinguish lung segments that are distal to the bronchus into which the bronchoscope is wedged. Furthermore, significant mixing of distal, alveolar and proximal bronchial compartments occurs. The mediators are usually diluted in the large volumes of physiological saline solution used in the washing, and some exchange with the blood compartment is inevitable. In contrast, induced sputum probably provides a more representative sample of several proximal airways, although with prolonged induction the distal parts can also be sampled, as evident from increased numbers of macrophages from the alveolar compartment. The comparison between bronchoscopy and induced sputum can be seen in Table 1.

**Table 1:** Comparison of the advantages and disadvantages of bronchoscopy and induced sputum

	Advantages	Disadvantages
Bronchoscopy	• Allows biopsy and BAL: samples can be obtained from mucosal tissue, and cells and mediators from the airway lumen	• Requires trained personnel and expensive equipment • Invasive
	• Provides information on structural changes (relating to epithelium, basement membrane and laminae)	• There is mixing of content from alveolar and bronchial compartments
	• Can be followed by immunohistochemistry, in situ hybridization, or electron microscopy	• Can be contaminated with blood
	• Can be followed by segmental allergen challenge • Allows the use of bronchial wash (cells for in vitro study)	• Biopsy samples can only be obtained from the larger airways and cell count reproducibility is low
Induced sputum	• Relatively non-invasive	• Risk of bronchoconstriction
	• Allows samples to be obtained from several proximal airways	• Success rate around 80%
	• Can be undertaken repeatedly	• Processing methods fairly laborious
	• Safe even in cases of severe disease	
	• No expensive equipment required	• Results not available immediately
	• Allows study of large patient populations	

*BAL: bronchoalveolar lavage*

## CLINICAL APPLICATIONS OF INDUCED SPUTUM

### Asthma

In clinical practice, it is difficult to assess airway inflammation and the effects of medication on such inflammation. Subjective assessment of symptoms is always difficult and has often been found to be unsatisfactory for monitoring asthma severity.^5^ Nonetheless, the regular use of peak flow measurements has been shown to improve asthma control, peak flow rates and diurnal peak flow relapse. Measurements of the levels of exhaled gases such as nitric oxide may be useful, but more data are needed to fully evaluate the importance of such markers in assessing airway inflammation in asthma, especially since nitric oxide can be produced in large amounts in paranasal sinuses and the stomach.^5^

Asthma is commonly associated with sputum eosinophilia. Up to 80% of corticosteroid-naive subjects and more than 50% of corticosteroid-treated subjects with currently symptomatic asthma have a sputum eosinophil count that is outside of the normal range. The validity of a high sputum eosinophil count for the identification of asthma is better than peak expiratory flow measurement.^6^

The short-term response to inhaled corticosteroids differs markedly according to the sputum eosinophil count, with little evidence of improvement in symptoms and airway responsiveness in subjects with a baseline sputum eosinophil count of less than 3%. These findings suggest that measuring the underlying airway inflammation might provide a better guide as to the need for corticosteroid treatment than assessment of functional abnormality.^6^

Occupational asthma is associated with an increase in sputum eosinophilia. There is some evidence that sputum eosinophil counts increase during workplace exposure in subjects with occupational asthma.^6^

One obvious application of sputum induction is to use sputum inflammatory indices to increase our understanding of complex relationships between inflammatory cells, mediators and cytokine mechanisms in asthma. The sputum fluid phase seems to be suitable for measuring eosinophil cationic protein, some cytokines and histamine. Assessment of airway inflammation using sputum could also be used for evaluating the effects of drugs on asthmatic airway inflammation and relating their anti-inflammatory effect to the effects on symptoms and disordered airway function.

### Chronic obstructive pulmonary disease

Chronic obstructive pulmonary disease is a clinical entity that is characterized by the presence of blockage or chronic limitation of the airflow that presents slow and irreversible progression. The origin of such alterations is the pulmonary combination of chronic bronchitis and emphysema. The pathophysiology of chronic obstructive pulmonary disease involves an inflammatory disorder characterized by neutrophilic inflammation in airway secretions, with the presence of macrophages and lymphocytes on airway tissue. Bronchoscopic investigations are often not possible due to disease severity.^7^ Thus, sputum induction is a valuable tool for pathophysiology studies. The sputum neutrophil count is usually high, and the neutrophil count can be correlated with a reduction in forced expiratory volume in one second (FEV_1_) and the rate of decline in FEV_1_, thus suggesting that neutrophilic airway inflammation is functionally important. Peleman et al. (1999) studied the cellular composition of induced sputum in chronic obstructive pulmonary disease and found marked sputum neutrophilia.^8^

Despite its nonspecific nature, the early inflammatory response to cigarette smoke is probably crucial to the development of subsequent tissue damage and disease in susceptible individuals. Neutrophils and macrophages can potentially produce large quantities of proteases, of which the various elastase enzymes have attracted the most attention as likely causes of the loss of elastic recoil and destruction of elastic fibers in the lung parenchyma. Indeed, lung specimens from patients with panlobular emphysema have a significantly decreased elastin content.^9^

Confalonieri et al. (1998) studied the effects of two months of treatment with inhaled beclomethasone dipropionate (1,500 μg/day) on bronchial inflammation in patients with stable, mild to moderate chronic obstructive pulmonary disease, by using sputum induction. They found that the number of neutrophils present in induced sputum samples decreased after treatment.^10^ Moreover, a short course of oral glucocorticoid therapy has been demonstrated to improve pulmonary function in some patients with chronic obstructive pulmonary disease, but not all.^11^

In a recent prospective trial, Borbeau et al. (1998) showed that, in a group of 140 chronic obstructive pulmonary disease patients, 19 (13.5%) responded to the two-week treatment with 40 mg prednisone daily. Response to treatment was defined as a 15% improvement in FEV_1._^12^ Also employing FEV_1_ to assess response, Mendella et al. (1982) showed that 17% of chronic obstructive pulmonary disease cases were considered responsive to a course of 32 mg/d methylprednisolone for two weeks.^13^

Furthermore, Brazilian authors studying sputum eosinophilia in smokers have found that eosinophilic inflammation can occur in smokers with or without chronic airflow limitation (chronic obstructive pulmonary disease) and that sputum eosinophilia may predict those patients who will benefit from steroid therapy.^14^

Fujimoto et al. (1999) investigated the influence of glucocorticoid in the reversibility of eosinophilic inflammation in patients with pulmonary emphysema. They found that the reversibility of airway obstruction following the treatment could be correlated with the eosinophil count in the induced sputum, and that the treatment significantly reduced eosinophil count and eosinophil mediators. In addition, patients who did not show improvement in FEV_1_, had lower baseline eosinophil counts.^11^

In conclusion, sputum assessment could be used as a screening test before deciding on long-term corticosteroid treatment in chronic obstructive pulmonary disease.

### Cough

Chronic cough is associated with predominant sputum neutrophilia, but up to 40% of subjects with cough have a sputum eosinophil count of more than 3%. Patients with cough and sputum eosinophilia exhibit an objective response to corticosteroid treatment that occurs in parallel with a treatment-associated fall in the sputum eosinophil count. In contrast, patients without sputum eosinophilia do not respond.^6^

### Tuberculosis

Pulmonary tuberculosis remains one of the most important health problems in the world.^15^ The World Health Organization recommends the detection of acid-fast bacilli in respiratory specimens as the initial approach to the diagnosis of tuberculosis.^16^ However, this method has low sensitivity and has little value in patients who cannot produce sputum spontaneously. In Brazil, with an estimated annual prevalence of 129,000 cases, approximately 22% of adult HIV-seronegative patients with suspected tuberculosis do not produce sputum spontaneously, or have negative acid-fast bacilli smears.^17^ Thus, the diagnosis of tuberculosis in these patients is difficult, and in most cases they are treated empirically on the basis of clinical and chest radiographic findings. However, empirical therapy may result in unnecessary cost and toxicity. Moreover, HIV-seropositive patients who do not produce sputum often undergo expensive and more invasive procedures.^17^ Thus, sputum induction is a valuable tool for diagnosing pulmonary tuberculosis.

Conde et al. (2000) compared sputum induction with fiberoptic bronchoscopy in the diagnosis of tuberculosis in a reference center in Rio de Janeiro, Brazil. They found that sputum induction is a safe procedure with high diagnostic yield and high agreement with the results from fiberoptic bronchoscopy, for the diagnosis of tuberculosis in HIV-seronegative and HIV-seropositive patients. In localities where fiberoptic bronchoscopy is not readily available, and as part of the work-up of suspected tuberculosis prior to bronchoscopy, induced sputum offers an alternative or additional approach to the diagnosis of sputum smear-negative tuberculosis, and would enhance diagnostic sensitivity in resource-poor areas.^17^ Anderson et al. (1995) compared sputum induction to fiberoptic bronchoscopy in the diagnosis of pulmonary tuberculosis in immunocompromised patients and found that sputum induction was well-tolerated, low-cost and provided the same, if not better, diagnostic yield compared with bronchoscopy in the diagnosis of smear-negative pulmonary tuberculosis.^18^

Bacteriological confirmation of pulmonary tuberculosis in infants and children remains difficult. Older children can produce or be induced to produce sputum. However, there are no reports of its use in infants or children younger than 3 years of age.

Gastric lavage is regarded as the standard procedure for obtaining specimens for staining and culture of *Mycobacterium tuberculosis* in younger children, because they swallow their sputum and do not expectorate. But sputum induction can be effectively performed and is well tolerated and safe, even in infants. Zar et al. (2000) compared induced sputum and gastric lavage for the isolation of *M. tuberculosis* in both HIV-infected and uninfected infants and children and found that induced sputum is better than gastric lavage.^19^ The bacteriological yield from sputum or gastric lavage in cases of pulmonary tuberculosis does not differ according to HIV status. The use of induced sputum should be considered as a first-line investigation in children suspected of having pulmonary tuberculosis, especially in circumstances in which a culture-confirmed diagnosis needs to be vigorously sought (such as when the source of the case is unknown, drug resistance is suspected, or cutaneous allergy occurs).^19^

### Cystic fibrosis

Cystic fibrosis is a hereditary disease of autosomal recessive transmission also known as mucoviscidosis or cystic fibrosis of the pancreas. The fundamental abnormality consists of the production of abnormal secretions from a variety of exocrine glands. It is chiefly a disease of infants and children, although adult cases are being recognized with greater frequency. There is no sex predominance. Involvement of the lungs usually is manifested clinically by recurrent chest infections (*Pseudomonas aeruginosa*, *Staphylococcus aureus*, *Haemophilus influenzae*) that are associated with wheezing, dyspnea, productive cough, and hemoptysis, as a result of bronchiectasis. Respiratory insufficiency and cor pulmonale develop frequently in the later stages of the disease. The lack of pancreatic enzymes results in poor digestion, particularly of fat.^20^ Inflammation begins at an early age, even in the absence of concomitant infection, and persists and progresses throughout life, ultimately leading to lung destruction. Quantitative measurements of infection and inflammation are therefore important in disease staging and new treatment evaluation.^21^

Each of the current techniques used for defining the microbiology and inflammatory response of the cystic fibrosis airway has notable limitations. Expectorated sputum provides an accurate measure of infection and inflammation in the lower airways, but many children with cystic fibrosis are unable to spontaneously expectorate sputum.^21^ Fiberoptic bronchoscopy with bronchoalveolar lavage is invasive, risky and costly. Serial bronchoalveolar lavages are particularly difficult to perform. Furthermore, lavage generally samples only one or two segments of the lung, thereby possibly limiting the detection of infection. Oropharyngeal cultures, commonly used in young children with cystic fibrosis who are not capable of expectorating, do not reliably predict the presence of lower airway pathogens, lack sensitivity for identifying *Pseudomonas aeruginosa* and *Staphylococcus aureus*, and provide no information about inflammation.^21^

In older children who cannot spontaneously expectorate sputum, induction can be a helpful diagnostic tool. Yet this diagnostic tool has received very little attention in cystic fibrosis. Recently, sputum induction was compared with spontaneously expectorated sputum and bronchoalveolar lavage in 10 adults with cystic fibrosis.^22^ Induced sputum was well tolerated by and preferred over broncho-alveolar lavage by all subjects, and resulted in larger sample volumes than for regular sputum samples.^22^ Induced sputum yielded similar cell numbers and similar detection rates for bacterial pathogens.^22^ Sputum induction appears to be a relatively safe, noninvasive means of obtaining airway secretions from subjects with cystic fibrosis, especially from those who do not normally produce sputum.^21^ Airway inflammation and infection are significantly increased in both non-expectorating and expectorating children with cystic fibrosis, in comparison with healthy children.^21^ Also, induced sputum samples appear to be comparable to spontaneously expectorated samples in describing both inflammation and infection in the cystic fibrosis airway.^21^ Induced sputum differs from spontaneous sputum by having a higher number of viable cells and less squamous cell contamination.^22^

### Lung Cancer

With regard to lung cancer, identification of early (or pre-symptomatic) lung cancer in smokers is considered the best strategy for preventing this disease.^5^ But cytological examination of sputum has been shown to lead to lung cancer detection at an earlier stage, thereby resulting in an improved five-year survival rate. Recent studies of sputum specimens and clinical data linking specimens to lung cancer outcomes may make it possible to determine molecular diagnoses of cancer several years before its clinical presentation. This has become possible through the use of tests to evaluate altered gene expression, including specific oncogene activation and tumor suppressor gene detection, as well as genomic instability and abnormal methylation. Such studies clearly indicate that good sputum samples ought to allow complicated genetic analysis to be performed, thus providing further impetus for considering the induced sputum technique as a tool for lung cancer screening.^5^

### *Pneumocystis Carinii* Pneumonia

*Pneumocystis carinii* pneumonia remains a significant cause of morbidity and mortality in HIV-infected individuals, causing clinically apparent pneumonia virtually exclusively in immunosuppressed patients. The clinical presentation is characterized by fever, shortness of breath, substernal tightness, and nonproductive cough. Especially in HIV-infected patients, the symptoms can be relatively mild and slowly progressive, which may delay diagnosis.^23^ Transbronchial biopsy and bronchoalveolar lavage have been shown to have 98 to 100% yield and 92 to 100% negative predictive value for the diagnosis of *Pneumocystis carinii* pneumonia. Although these are considered to be the gold standard, they still entail some morbidity and are relatively expensive procedures.^24^ Thus, induced sputum may have a role in diagnosing *Pneumocystis carinii* pneumonia.

It was reported in the mid-1980s that the examination of sputum induced by the inhalation of hypertonic saline solution was frequently diagnostic for *Pneumocystis carinii* pneumonia.^1^ Since then, this diagnostic method has generally become the first employed when *Pneumocystis carinii* pneumonia is suspected.^25^ Kirsch et al. (1990), studying 62 patients with possible aids-associated *Pneumocystis carinii* pneumonia to determine the diagnostic usefulness of sputum analysis, found that sputum analysis is a sensitive, specific, rapid and low-cost technique for the diagnosis of *Pneumocystis carinii* pneumonia.^24^

## METHODS FOR SPUTUM INDUCTION AND PROCESSING

### Induction

Ultrasonic nebulizers are recommended for sputum inducing since other nebulizers do not usually have sufficient saline aerosol output. Spirometry is necessary to assess the baseline airway caliber and avoid excessive bronchoconstriction during saline inhalation. Spirometers are preferable to peak flow meters because of the greater sensitivity of FEV_1_ in detecting induced bronchoconstriction. Sputum induction requires a high degree of cooperation from the patient. The procedure should be conducted by an experienced technician under the supervision of an experienced physician.^26^

Because hypertonic saline causes bronchoconstriction in asthmatic subjects,^27^ pre-treatment with a short-acting beta-2 agonist is recommended as the standard procedure.^2,28^

Salbutamol, usually 200-400 mmg, i.e. 2-4 puffs from a standard metered-dose inhaler, has generally been used for pretreatment.^26^

The concentration of the saline used for sputum induction has ranged from 0.9 to 7%.^28-30^ The concentration may be changed during the procedure, starting with 3% and subsequently increasing to 4 or 5%.^2,31^ Hyper-tonic saline solution is reportedly more effective than isotonic saline in inducing sputum.^32^

**Figure 1:**
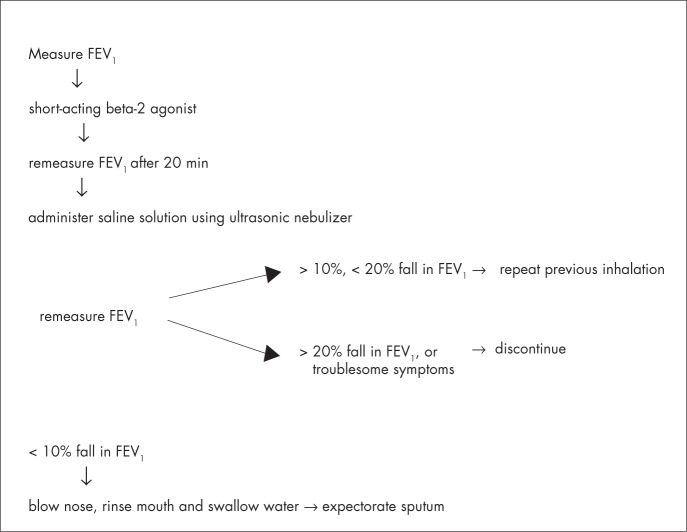
Method for sputum induction.

Different compartments of the respiratory tract are sampled at different time-points during induction, i.e. central airways are sampled early, whereas peripheral airways and alveoli are sampled later. Shorter inhalation times (15-20 min) appear to have feasibility and success rates that are similar to those of longer inhalation times (30 min). The consensus is to use cumulative nebulization duration of 15-20 minutes.^26^ The procedures for induction can be seen in Figure 1.

### Sputum Processing

It is recommended that sputum be processed as soon as possible or within two hours, in order to ensure optimum cell counting and staining.^33,34^ Complete homogenization is important and can be achieved by the use of dithiothreitol (DTT). Cells that are incompletely released from mucus tend to stain darkly, making correct identification difficult. DTT (0.1%) has been shown to be more effective for dispersing cells than phosphate-buffered saline (PBS), and has no adverse effects on cell counts. The volume of mucolytic agents used during the processing of all the expectorated sputum, although fixed at 1:1, is variable in relation to the sputum/saliva ratio, which is an unknown.^34,35^ Filtration through a 48-μm nylon mesh is commonly used to remove mucus and debris, and is strongly recommended.^36^

Centrifugation is necessary to separate sputum cells from the fluid phase. Centrifugal forces used in studies to date have ranged from 300 to 1,500 xg and the duration of centrifugation from 5 to 10 minutes. The total cell count is performed manually using a hemocytometer, and cell viability is determined by the trypan blue exclusion method.^34,37^ Fluid phase storage temperatures used have ranged from −20 to −70° C.^36^

**Figure 2:**
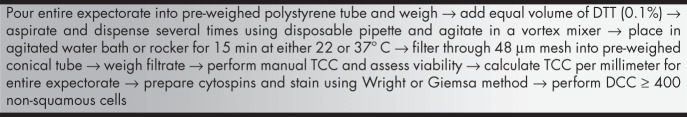
Sputum processing method

Preparation of cytospins with an optimum number of cells (40-60 x 10^3^ cells) provides a more accurate estimate of cell distribution than smears. Cytocentrifugation speeds range from 10 to 51 xg (using a cytocentrifuge), with the most common conditions being 22 xg for 6 minutes.^37,38^ Cytospin staining for differential cell counts can be achieved using either Wright or Giemsa staining. The differential cell count is determined by counting a minimum of 400 non-squamous cells, and is reported as the relative numbers of eosinophils, neutrophils, macrophages, lymphocytes and bronchial epithelial cells, expressed as a percentage of total non-squamous cells. The squamous cell percentage should always be reported separately.^36^ The processing procedures can seen be in Figure 2.

*TCC: total cell count; DCC: differential cell count; DTT: dithiothreitol.*

### Objective quantitative analysis of cells in sputum

Manual differential counting of sputum cytospins is tedious to perform and, although based on objective morphological criteria, observer variability makes more objective assaying desirable. The laser scanning cytometer is a novel microscope-linked and computer-operated instrument that measures fluorescence and optically scans cells labeled with fluorescent probes on a microscope slide.^39^

## EXAMPLE OF PROTOCOL USED IN BRAZIL

The protocol for inducing and processing sputum used by the Pulmonary Division, Medical School of Ribeirão Preto, Universidade de São Paulo, is as follows.

### Nebulizer

**Figure 3:**
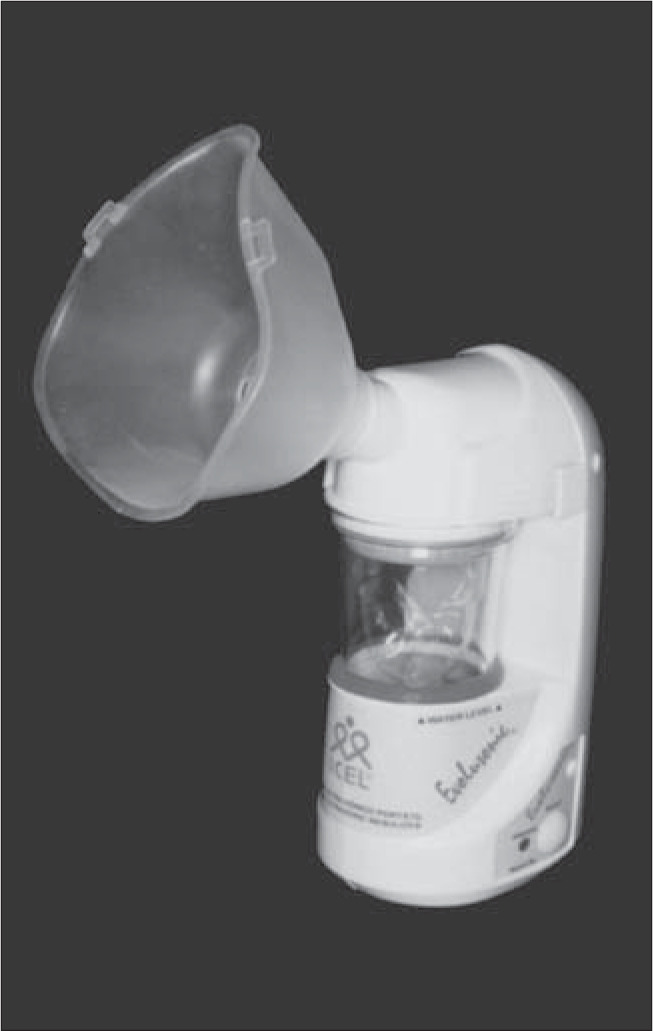
Icel ultrasonic nebulizer (model Evolusonic 1000BR).

**Figure 4:**
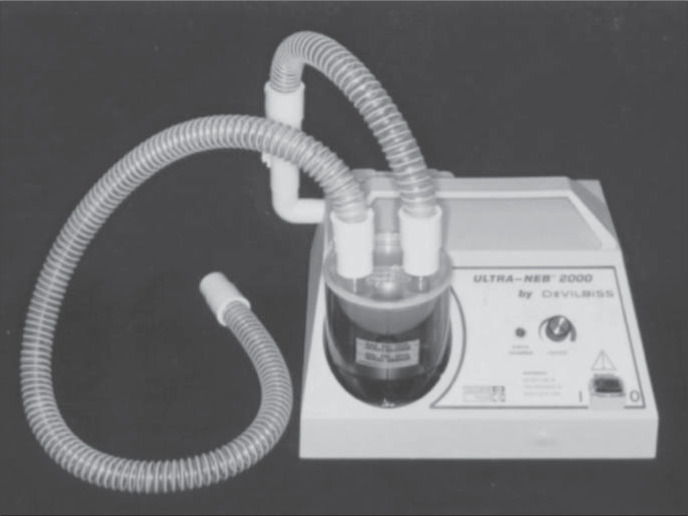
DeVilbiss ultrasonic nebulizer (model Ultra-Neb 2000).

We compared two ultrasonic nebulizers for sputum induction, one Brazilian and the other imported, in terms of safety (bronchospasm risk) and yield (sputum volume and cell numbers).^40^ Patients with mild or moderate asthma formed two groups that underwent sputum induction by inhalation of NaCl 4.5% for 20 minutes. Peak flow measurements were done before induction and every five minutes during induction. Group 1 used the Icel nebulizer (Figure 3) (Evolusonic 1000BR, Icel, São Paulo, SP, Brazil), a low-output nebulizer whose measured output rate was 1 ml/min (n = 9). Group 2 used the DeVilbiss nebulizer (Figure 4) (Ultra-Neb 2000, DeVilbiss-Sunrise Medical, Somerset, Pennsylvania, United States of America), a high-output nebulizer with a rate of 2.5 ml/min (n = 9).

The results can be seen in Table 2.

**Tabela 2:** Comparison between two different ultrasonic nebulizers in sputum induction (n = 18)

	Icel*	DeVilbiss**	P (t test)
Fall in peak flow	11.65 ± 3.8	19.9 ± 3.6	0.14
Sputum volume (ml)	4.34 ± 1.2	13.2 ± 3.2*	0.02
Total cell count (million)	4.4 ± 1.0	14.6 ± 4.3*	0.03
Cells/ml (million)	1.64 ± 0.3	1.33 ± 0.3	0.49
Cell viability (%)	82.3 ± 3.5	83.4 ± 2.7	0.80

* *Evolusonic 1000BR, Icel, Brazil;*

** *Ultra-Neb 2000, DeVilbiss-Sunrise Medical, United States of America.*

We concluded that the much greater sputum induction seen in Group 2 might be a consequence of the higher output rate provided by the nebulizer used in that group (DeVilbiss). On the basis of this conclusion, we have been utilizing the high-output nebulizer (DeVilbiss) for routine induction. However, in cases of severe asthma, we may prefer low output so as to decrease the risk of bronchospasm. Moreover, the high cost of the imported equipment may lead us to use the Brazilian nebulizer, which is also effective in inducing sputum.

### Induction Method

Measure peak flow. Apply 2 to 4 puffs of salbutamol (200-400 μg). After 20 minutes, measure peak flow and calculate the critical peak flow (fall of 10%). Administer NaCl 4.5%, using a ultrasonic nebulizer like DeVilbiss. Interrupt this every 5 minutes, to discard saliva and collect sputum in a specific tube. Total induction time is 20 minutes, or less if peak flow falls to the critical value.

### Processing Method

Weigh the sputum. Add an equal volume of DTT. Aspirate and dispense several times using a disposable pipette, and agitate in a vortex mixer. Agitate in a water bath at 37° C (150 cycles/min) for 15 minutes, with aspiration every 5 minutes for homogenization. Centrifuge at 750 g for 10 minutes, with medium acceleration/deceleration. Remove the supernatant using a micropipette and store it at −70° C. Resuspend the cell pellet in 1 ml of PBS. Determine cell viability by means of the trypan blue exclusion method in a Newbauer chamber (non-viable cells are stained blue). Perform the total count in the Newbauer chamber after Turk staining. Adjust the cell solution to 0.5 million cells/ml of PBS. Prepare cytospins: 75 μl/cups to spin for 60 seconds at 1,000 rpm (in cytocentrifuge). Stain the slides using the May-Grunwald and Giemsa methods.

## FUTURE DIRECTIONS

The identification of biomarkers that allow early diagnosis, monitoring and optimization of lung disease therapy is one of the most ambitious goals in respiratory medicine. The induced sputum technique allows sampling of the airways in a noninvasive fashion and thus offers a unique opportunity for identifying biomarkers of potential clinical use in respiratory medicine. It is hoped that, in the future, induced sputum will provide clinicians with useful markers that can be used routinely for performing more accurate and, ideally, more rapid determination of disease pheno-types in many lung diseases.

The hope is that the induced sputum technique will provide a simple and cost-effective tool for monitoring airway inflammation in the clinical setting, an approach that was precluded by previous techniques such as bronchial biopsy and bronchoalveolar lavage. In addition, since the induced sputum technique enables regular monitoring of inflammation, it will be of great help in assessing the anti-inflammatory potential of new treatments.
